# Untargeted Serum Metabolomics Reveals Metabolic Disturbances in Children with MASLD: A Preliminary Study

**DOI:** 10.3390/ijms27198742

**Published:** 2026-09-30

**Authors:** Ewa Kowalska-Ciszek, Julia Zelkowska, Wojciech Jańczyk, Katarzyna Patla, Anna Siejka, Joanna Klimiuk-Balas, Magdalena Zielińska, Anna Świąder-Leśniak, Joanna Godzien, Dominik Cysewski, Agnieszka Ochocińska, Mieczysław Litwin, Michał Ciborowski, Piotr Socha

**Affiliations:** 1 Department of Clinical Biochemistry, The Children’s Memorial Health Institute, 04-730 Warsaw, Poland; katarzynapatla@gmail.com (K.P.); a.siejka@ipczd.pl (A.S.); j.klimiuk-balas@ipczd.pl (J.K.-B.); magdalena.zielinska@ipczd.pl (M.Z.); a.ochocinska@ipczd.pl (A.O.); 2 Metabolomics and Proteomics Laboratory, Clinical Research Centre, Medical University of Bialystok, 15-276 Bialystok, Poland; julia.zelkowska@umb.edu.pl (J.Z.); joanna.godzien@umb.edu.pl (J.G.); dominik.cysewski@umb.edu.pl (D.C.); michal.ciborowski@umb.edu.pl (M.C.); 3 Department of Gastroenterology, Hepatology and Nutrition Disorders, The Children’s Memorial Health Institute, 04-730 Warsaw, Poland; w.janczyk@ipczd.pl; 4 Department of Anthropology, The Children’s Memorial Health Institute, 04-730 Warsaw, Poland; a.swiader-lesniak@ipczd.pl; 5 Department of Nephrology and Arterial Hypertension, The Children’s Memorial Health Institute, 04-730 Warsaw, Poland; m.litwin@ipczd.pl; 6 Department of Medical Biochemistry, Medical University of Bialystok, 15-276 Bialystok, Poland

**Keywords:** pediatric MASLD, untargeted metabolomics, 12α-hydroxy-3-oxocholadienic acid, bile acid metabolism, acylcarnitine

## Abstract

Pediatric metabolic dysfunction-associated steatotic liver disease (MASLD) has emerged as a major public health concern. The pediatric form of MASLD differs from the adult phenotype in its pathophysiology and exhibits distinct histopathological characteristics. This study aimed to characterize serum metabolomic alterations associated with pediatric MASLD and to identify metabolic signatures that may provide further insight into the disease phenotype. To this end, untargeted metabolomic analysis was performed on serum samples from 21 pediatric MASLD patients and 22 healthy controls using liquid chromatography coupled with high-resolution quadrupole time-of-flight mass spectrometry (LC-MS/QTOF). Univariate statistical analysis was performed using the Mann–Whitney U-test with false discovery rate (FDR) correction, while multivariate analysis employed principal component analysis and orthogonal partial least squares discriminant analysis (OPLS-DA). Multivariate analysis in the positive ionization mode demonstrated clear separation between the MASLD and control groups (R^2^Y = 0.972, Q^2^ = 0.823, CV-ANOVA *p*-value = 2.387 × 10^−10^). A total of 46 annotated metabolites differed significantly between the groups. The most pronounced alterations involved bile acid and sterol metabolism, including increased 12α-hydroxy-3-oxocholadienic acid and decreased conjugated primary bile acids, including taurine and glycine conjugates. In addition, increased levels of free carnitine and short-chain acylcarnitines, together with alterations in free and oxidized fatty acids, suggested disturbances in fatty acid metabolism. The metabolomic profile also revealed broad disruption of glycerophospholipid metabolism, characterized primarily by decreased lysophosphatidylcholines and lysophosphatidylethanolamines. In conclusion, the metabolomic profile of pediatric MASLD indicates pronounced systemic metabolic alterations, primarily involving lipid and fatty acid metabolism, bile acid and sterol metabolism, and amino acid-related pathways. The coordinated pattern of these changes suggests alterations across multiple interconnected metabolic processes, highlighting the complex metabolic phenotype of pediatric MASLD. The identified metabolic signatures provide a basis for further investigation and warrant validation in larger, independent cohorts to determine their biological and potential clinical relevance.

## 1. Introduction

Pediatric metabolic dysfunction-associated steatotic liver disease (MASLD, formerly known as non-alcoholic fatty liver disease, NAFLD) has emerged as a major public health concern, driven by the global obesity epidemic, sedentary lifestyles, and complex mechanisms including insulin resistance, lipotoxicity, oxidative stress, and gut dysbiosis [1,2,3,4,5,6,7,8,9].

While clinical features often mirror those of adults, pediatric MASLD presents distinct pathophysiological and histopathological characteristics, such as portal-based inflammation and age-specific molecular signatures. These distinctions may result from differing underlying mechanisms, such as developmental factors, perinatal influences, and unique immunometabolic interactions [2,4,10,11,12]. Moreover, male sex, ethnicity, and genetic predispositions are strongly associated with pediatric MASLD, with gender-specific patterns observed in metabolic markers and liver enzyme levels [13].

The pathophysiology of MASLD extends far beyond the simple accumulation of fat within hepatocytes. The specific composition of the lipidome, encompassing a vast array of molecular species from triglycerides and free fatty acids to complex phospholipids and sphingolipids, plays a direct role in the progression from benign steatosis to the inflammatory and fibrotic state [14,15].

Moreover, disruptions in the gut-liver axis significantly drive pediatric MASLD progression. Intestinal dysbiosis and increased permeability allow microbial products (e.g., lipopolysaccharide, LPS) to translocate into the liver, triggering severe inflammation and altering critical signaling pathways, including those governed by sterol lipids and bile acids. This environment generates reactive oxygen species (ROS), which activate hepatic stellate cells and accelerate fibrogenesis [5,12,16].

Management of pediatric MASLD remains a significant clinical challenge due to the lack of approved pharmacotherapies. Consequently, pharmacological research has intensified, initially evaluating repurposed metabolic agents such as insulin sensitizers and GLP-1 receptor agonists. A pivotal frontier in targeted therapy is the Ileal Farnesoid X Receptor (FXR) axis, which regulates bile acid homeostasis and the FGF19 pathway. Second-generation non-steroidal agonists (e.g., Cilofexor) offer improved tolerability but limited monotherapy efficacy. Ultimately, addressing the complex pathophysiology of pediatric MASLD requires moving beyond monotherapy toward combination strategies [15,16,17,18,19].

Moreover, despite its severity, pediatric MASLD remains underdiagnosed due to the limitations of current non-invasive diagnostic tools, with liver biopsy remaining the invasive gold standard. Future success depends on the validation of sensitive, non-invasive biomarkers for patient stratification and a broader investigation into signaling crosstalk between the microbiome, bile acids, and nuclear receptors to identify synergistic therapeutic targets. Addressing this gap requires advanced analytical techniques. High-resolution untargeted metabolomics utilizing LC-MS/QTOF technology provides a sensitive platform for comprehensive metabolic profiling [20,21,22,23,24]. By mapping these specific biomolecular signatures—ranging from bile acid dysregulation to phospholipid depletion—this approach holds significant promise for discovering non-invasive biomarkers, allowing for earlier detection and a better understanding of the gut-liver axis mechanisms driving pediatric MASLD [12,25,26].

## 2. Results

### 2.1. Untargeted Metabolomic Analysis of Serum Samples

**Figure 1:**
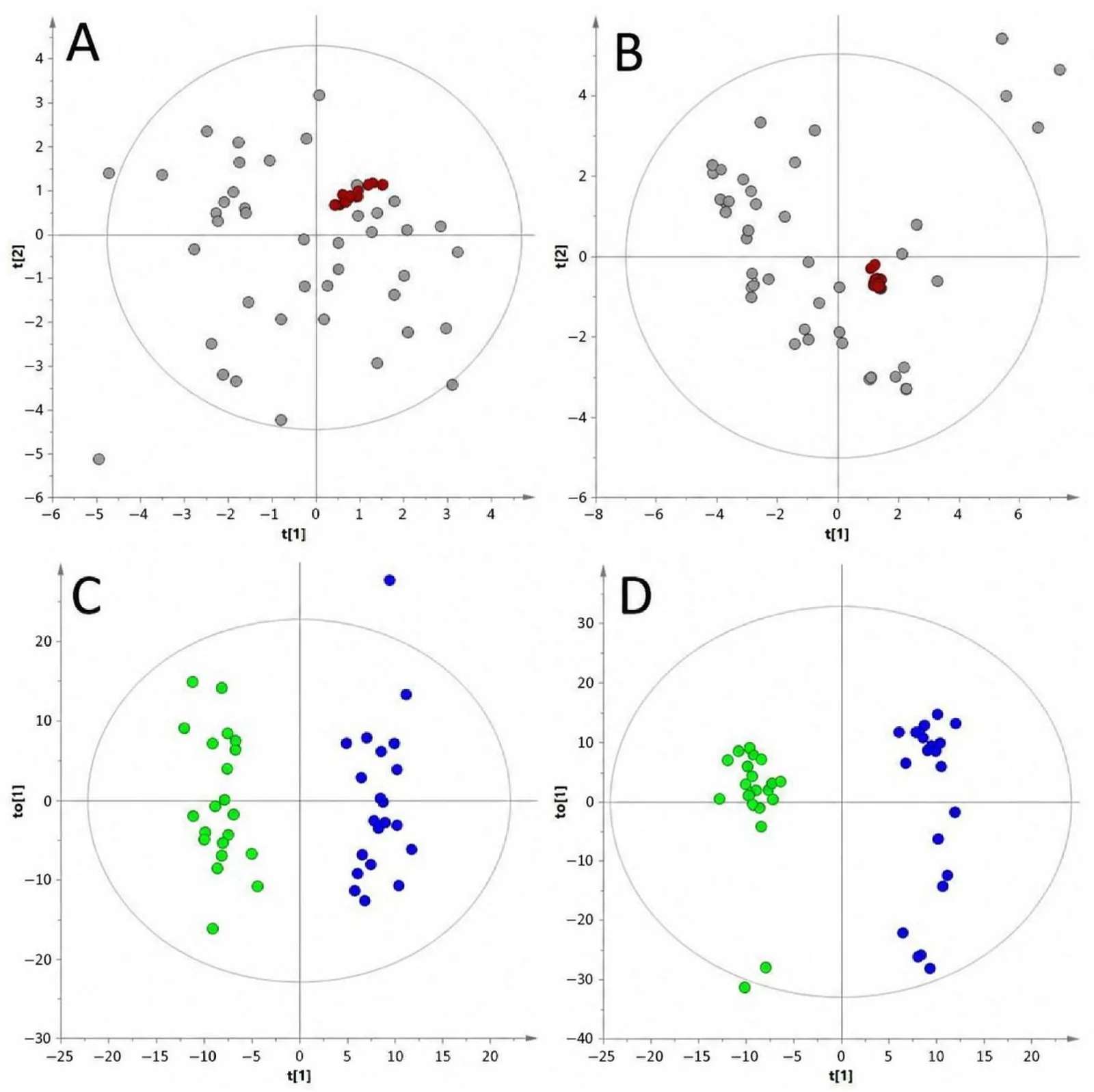
PCA and OPLS-DA score plots of the serum metabolomic profiles of MASLD and controls. Panels (**A**) (negative ion mode) and (**B**) (positive ion mode) show the classification of QC samples (red dots) on PCA models; grey dots represent study samples. Panels (**C**) (negative ion mode, R^2^Y = 0.953, Q^2^ = 0.709, CV-ANOVA *p*-value = 3.880 × 10^−8^) and (**D**) (positive ion mode, R^2^Y = 0.972, Q^2^ = 0.823, CV-ANOVA *p*-value = 2.387 × 10^−10^) show the classifications of MASLD (green dots) and control (blue dots) samples on OPLS-DA models.

Serum samples from children with MASLD and controls were analysed using untargeted LC-MS profiling. The obtained data were filtered and subjected to a Quality Assurance procedure. As a result, two datasets were obtained, containing 891 metabolic features in positive ionization mode and 961 in negative ionization mode, respectively. Based on those data, principal component analysis (PCA) was performed to check the classification of the quality control (QC) samples. Close clustering of QCs on the obtained PCA models (Figure 1A,B) indicates the system’s stability and performance during analyses. To illustrate the classification of the studied samples based on the obtained serum metabolomic profiles, an orthogonal partial least squares discriminant analysis (OPLS-DA) was performed. Figure 1 shows clear (panel C) and adequate (panel D) separation of MASLD and control samples based on data obtained from negative and positive ion modes, respectively.

Univariate statistical analysis resulted in 165 significant metabolic features discriminating the MASLD group from the control group in negative ion mode and 228 in positive ion mode. Metabolite annotation yielded 52 annotated discriminatory features in negative ion mode and 30 in positive ion mode. All annotations were assigned MSI Level 2 confidence [27]. Details regarding annotation are provided in Supplementary Table S1. Among all annotated metabolites from both polarity modes, 46 were unique. The metabolites that differed significantly between the study groups are listed in Table 1. For these metabolites, the relative standard deviations (RSDs) of signal intensities in QC samples are presented in Supplementary Table S1.

**Figure 2:**
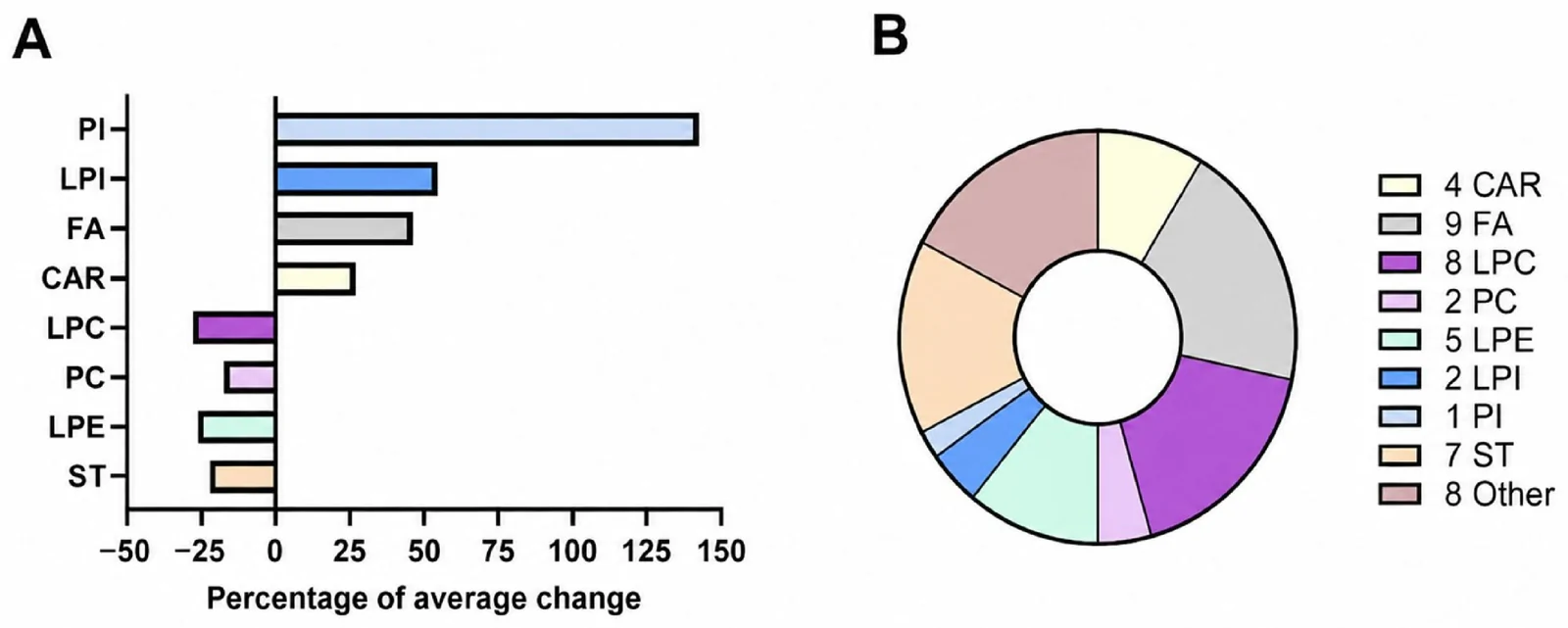
Panel (**A**) illustrates the percentage of the averaged change between the MASLD and control group across different lipid classes. A positive value means that an abundance of a particular group of metabolites was higher, whereas a negative value indicates that abundance was lower in the MASLD group vs. the control group. Panel (**B**) illustrates the number of lipids classified into individual classes that differentiate the studied groups. The number preceding each group abbreviation indicates the number of metabolites in that class (for example, 4 CAR denotes four carnitines that significantly differentiate the studied groups). Abbreviations: PI: phosphatidylinositol; LPI: lysophosphatidylinositol; FA: fatty acids; CAR: carnitine; LPC: lysophosphatidylcholine; PC: phosphatidylcholine; LPE: lysophosphatidylethanolamine; ST: sterol lipids.

The majority of chemically annotated metabolites were glycerophospholipids (glycerophosphocholines and glycerophosphoethanolamines) and were downregulated in the MASLD group compared with the control group. The exceptions were glycerophosphoinositols, which were upregulated in the MASLD group compared with the control group. Moreover, sterol lipids were predominantly downregulated in the MASLD group compared with the control group. In addition, fatty acids and carnitines were upregulated in the MASLD group compared with the control group. Among other metabolites, creatinine, malic acid and tryptophan were found to be downregulated in the MASLD group compared with the control group, while creatine, indoline, myristoylglycine, uric acid and 12α-hydroxy-3-oxocholadienic acid were found to be upregulated in the MASLD group compared with the control group. The percentage change in median values between the MASLD and control groups was calculated for each metabolite, and the mean percentage change was then determined for each annotated lipid class, as presented in Figure 2A. The distribution of metabolites differentiating MASLD patients from controls by chemical class is shown in Figure 2B.

### 2.2. Metabolic Pathway Analysis

**Figure 3:**
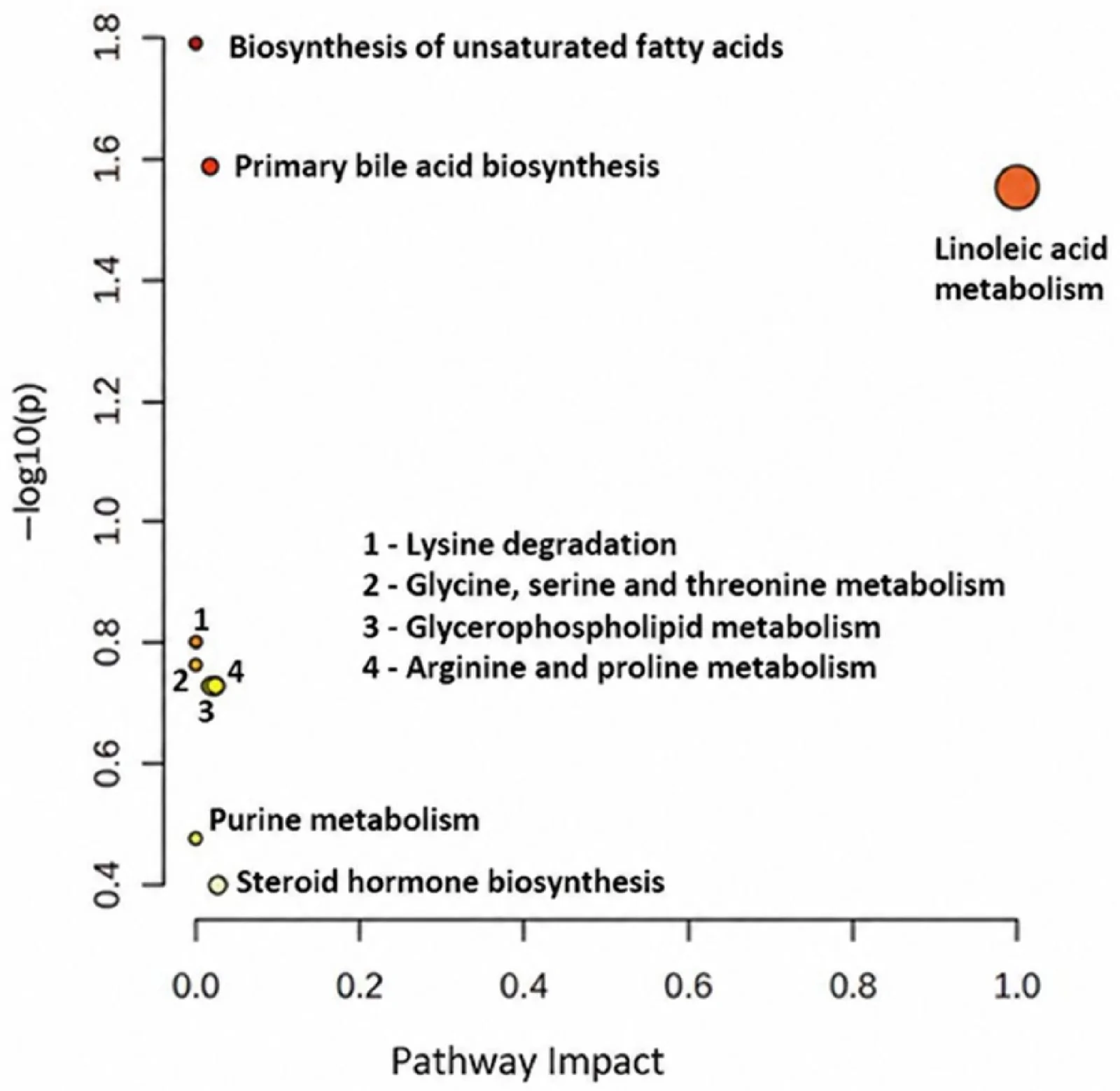
Metabolic pathway analysis based on serum samples of patients with MASLD compared to the control group.

To determine whether the metabolic profiles discriminating children with MASLD from the control group were associated with specific metabolic pathways, a pathway analysis was performed using MetaboAnalyst 6.0.13 with the Homo sapiens pathway library. The calculated *p*-value was established based on pathway enrichment analysis, whereas the pathway impact value was based on pathway topology analysis. As shown in Figure 3, Linoleic acid metabolism had the highest pathway impact, whereas the biosynthesis of unsaturated fatty acids and primary bile acid biosynthesis pathways were among the most significantly enriched pathways in MASLD.

## 3. Discussion

The provided dataset, derived from untargeted metabolomics by Liquid Chromatography–Mass Spectrometry (LC-MS) analysis, summarizes the median percentage change observed in 46 annotated metabolites in children with MASLD compared with controls. The data reveal significant alterations across several metabolic pathways, primarily related to amino acid derivatives, lipid metabolism (fatty acyls and phospholipids), and sterol/bile acid metabolism. The most striking metabolomic shifts observed in this cohort involve molecules linked to the bidirectional communication between the gut and the liver.

### 3.1. Sterol Lipids and Bile Acid Dysregulation

Several conjugated primary bile acids showed reduced relative abundances, which may indicate alterations in enterohepatic bile acid metabolism and recirculation. In contrast, some other sterol-related metabolites showed increased relative abundances. The observed changes included:

- ST 24:1;O3;Tau (a taurine conjugate) decreased by 62.47%;
- Glycine conjugates showed significant depletion: ST 24:1;O3;Gly decreased by 54.55%, and ST 24:1;O4;Gly decreased by 54.82%;
- Glycochenodeoxycholic acid 7-sulfate decreased sharply by 60.76%.
- The metabolite 12α-hydroxy-3-oxocholadienic acid showed the second-highest increase in the dataset (+118.58%). Additionally, ST 22:1;O3;S was increased by 84.77%.

**Figure 4:**
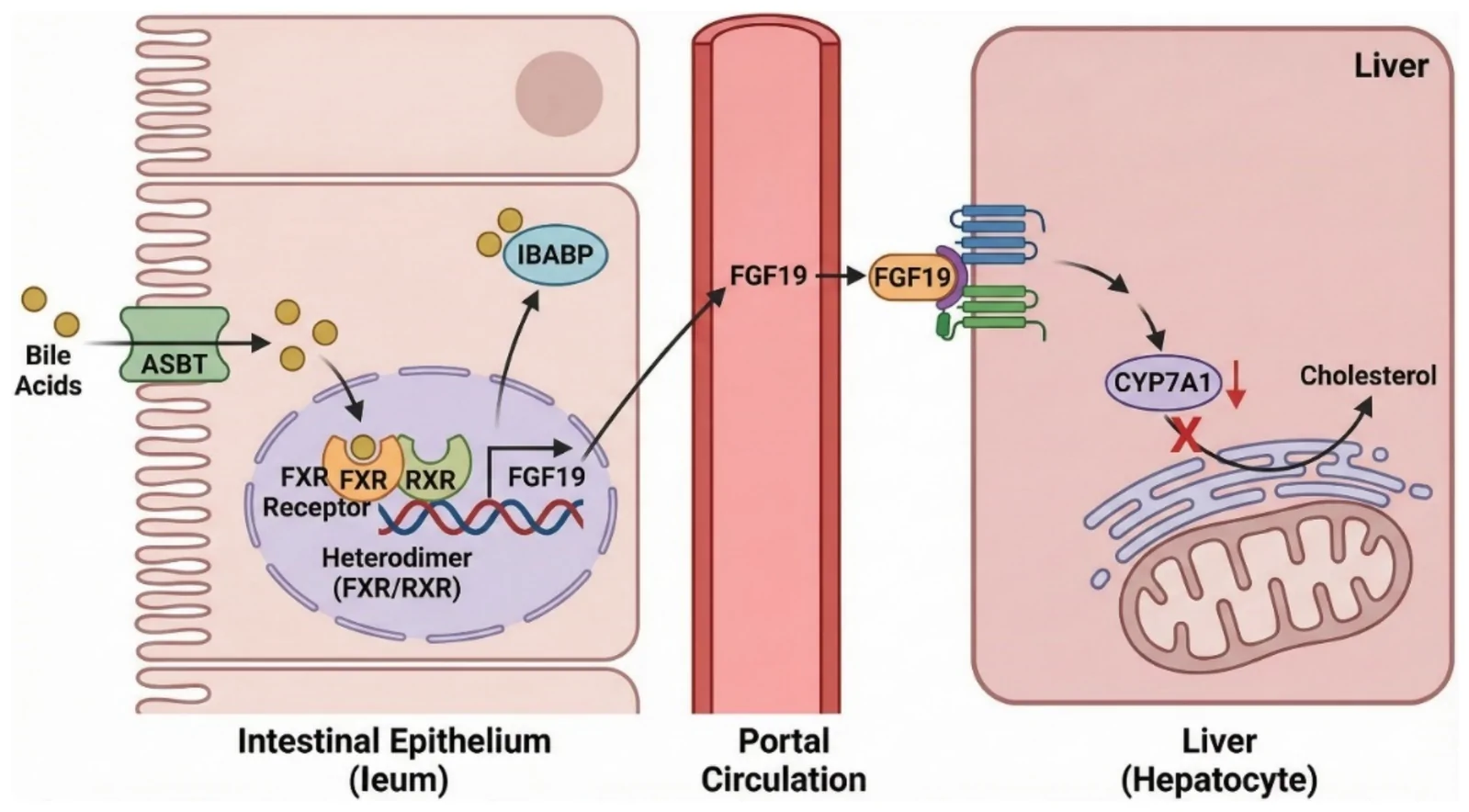
Physiological Regulation of the Gut-Liver Axis via the Canonical Ileal FXR-FGF19 Signaling Pathway. Abbreviations: ASBT: Apical Sodium-dependent Bile acid Transporter; FXR: Farnesoid X Receptor; RXR: Retinoid X Receptor; IBABP: Ileal Bile Acid Binding Protein; FGF19: Fibroblast Growth Factor 19; CYP7A1: cholesterol-7-α-hydroxylase.

Bile acids (BAs) are synthesized and conjugated in the liver, secreted into bile, and subsequently modified by intestinal microbes through deconjugation and conversion to secondary BAs (Figure 4).

The lower serum abundances of several conjugated BAs observed in our cohort are consistent with altered enterohepatic BA homeostasis but do not identify the initiating mechanism. Reduced serum glycine-conjugated bile acid concentrations have also been reported in children with nonfibrotic NAFLD [28]. Potential upstream determinants include changes in hepatic BA synthesis and conjugation, transporter-mediated hepatic uptake and biliary secretion, and intestinal reabsorption. The liver-spleen axis provides an additional immunometabolic framework in which splenic immune activation and cytokine release may influence hepatic metabolism [29]. Experimental evidence that inflammatory signaling can modify hepatic farnesoid X receptor (FXR) signaling and the expression of BA transporters, including sodium taurocholate cotransporting polypeptide (NTCP) and bile salt export pump (BSEP), supports the biological plausibility of such regulation [30,31]. However, neither splenic involvement nor the direction of changes in FXR signaling or transporter activity can be inferred from our serum metabolomic profiles.

Within this framework, host-mediated changes in the BA pool reaching the intestine could precede microbial adaptation, with dysbiosis emerging as a downstream consequence. This provides a mechanistic alternative to primary dysbiosis initiating BA dysregulation. Nevertheless, host and microbial BA metabolism are interdependent, and our cross-sectional study cannot establish their temporal or causal sequence. Serum abundances alone do not establish intestinal BA availability or the direction of changes in ileal FXR-fibroblast growth factor 19 (FGF19) signaling and hepatic CYP7A1 regulation; these pathways were not directly assessed in the present study [32].

**Figure 5:**
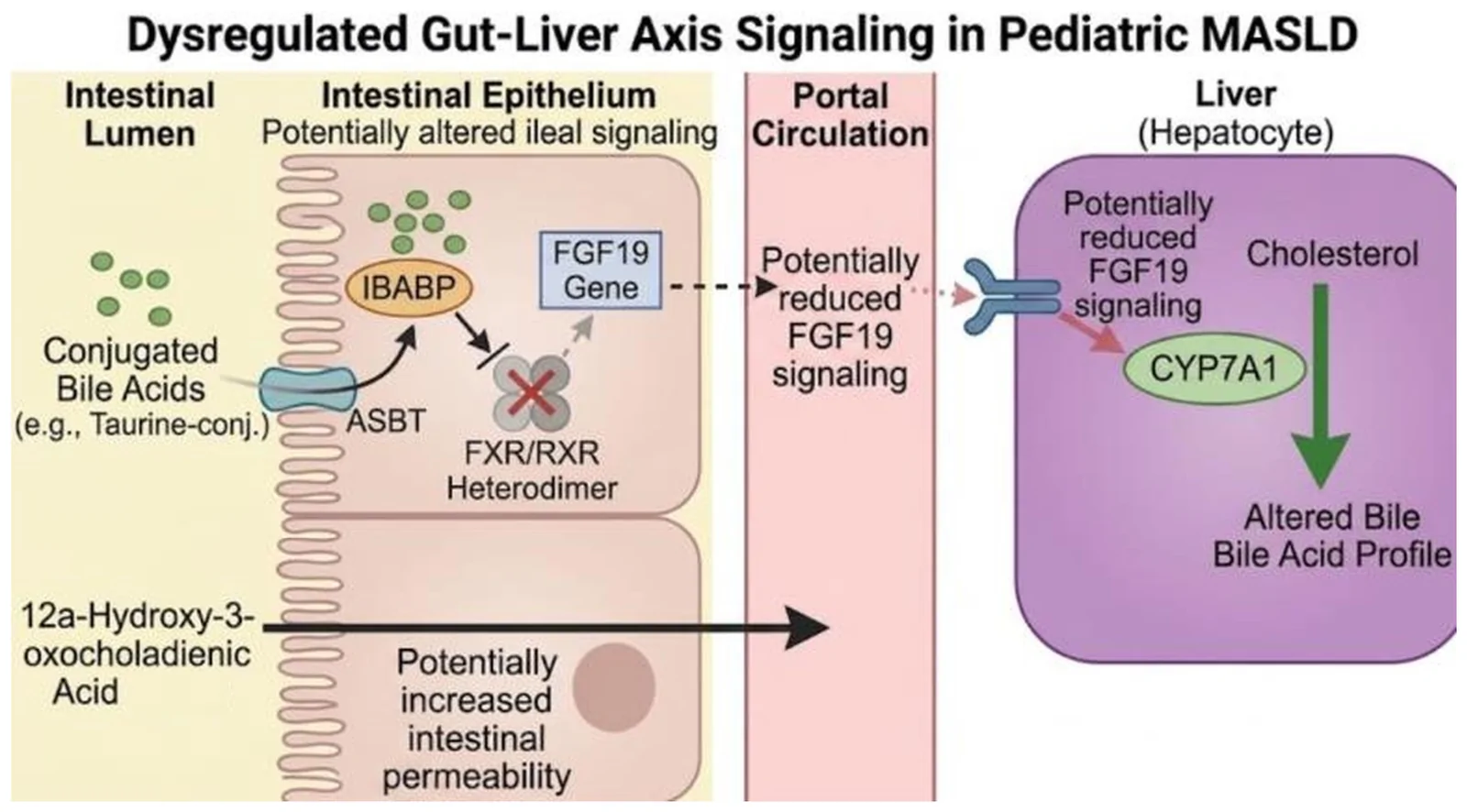
Hypothesized gut-liver axis mechanisms potentially associated with the bile acid alterations observed in pediatric MASLD. The proposed changes in FXR–FGF19 signaling, intestinal permeability, and CYP7A1 regulation were not directly assessed in the present study and are presented as a conceptual framework requiring further experimental validation. Serum metabolite abundances do not establish intestinal luminal concentrations or the direction of causality. Upstream changes in hepatic BA synthesis, conjugation, and transport, potentially influenced by liver-spleen immune signaling, provide an alternative explanation for the observed serum BA alterations and could precede microbial adaptation.

The metabolite 12α-hydroxy-3-oxocholadienic acid showed the second-largest increase in the dataset, while ST 22:1;O3;S was also increased. Previous studies in experimental models of colitis have associated 12α-hydroxy-3-oxocholadienic acid with alterations in intestinal barrier function, microbiota homeostasis, and inflammatory responses [33]. The increased circulating level of this metabolite observed in our cohort raises the possibility that it may be associated with alterations in gut-liver axis metabolism (Figure 5).

Additional context is provided by transcriptomic studies in pediatric fatty liver disease, in which peripheral blood mononuclear cells (PBMCs) showed molecular responses to microbial and fecal stimuli. Exposure to fecal extracts, containing a complex mixture of luminal metabolites and bile acids, induced pro-inflammatory responses, including increased IL-1β production [34]. However, these findings do not establish a specific role for 12α-hydroxy-3-oxocholadienic acid in these responses. Its potential as a candidate marker of altered gut–liver axis metabolism warrants further investigation, as the present data cannot determine whether its increased circulating level reflects changes in intestinal permeability, gut microbial metabolism, hepatic metabolism, or other processes. Its relationship with intestinal barrier integrity and systemic inflammation therefore requires direct experimental validation.

### 3.2. Lipid and Acylcarnitine Alterations in Pediatric MASLD

The metabolomic profile associated with MASLD was also characterized by alterations in metabolites related to lipid and fatty acid metabolism. In particular, changes in free carnitine, short-chain acylcarnitines, free fatty acids and oxidized fatty acid species were observed [14]. These alterations may provide insight into changes in fatty acid utilization and energy metabolism; however, they should be interpreted as metabolic associations rather than direct evidence of mitochondrial dysfunction.

Free carnitine and the detected short-chain acylcarnitines showed higher relative abundances in children with MASLD than in controls. Carnitine plays an essential role in the transport of long-chain fatty acids into mitochondria, while altered short-chain acylcarnitine levels have been associated with changes in fatty acid oxidation and mitochondrial energy metabolism. The observed increases included free carnitine (CAR 0:0, +18.21%), as well as several short-chain acylcarnitines, including CAR 2:0 (+24.37%), CAR 3:0 (+34.25%), and CAR 5:0 (+30.84%).

**Figure 6:**
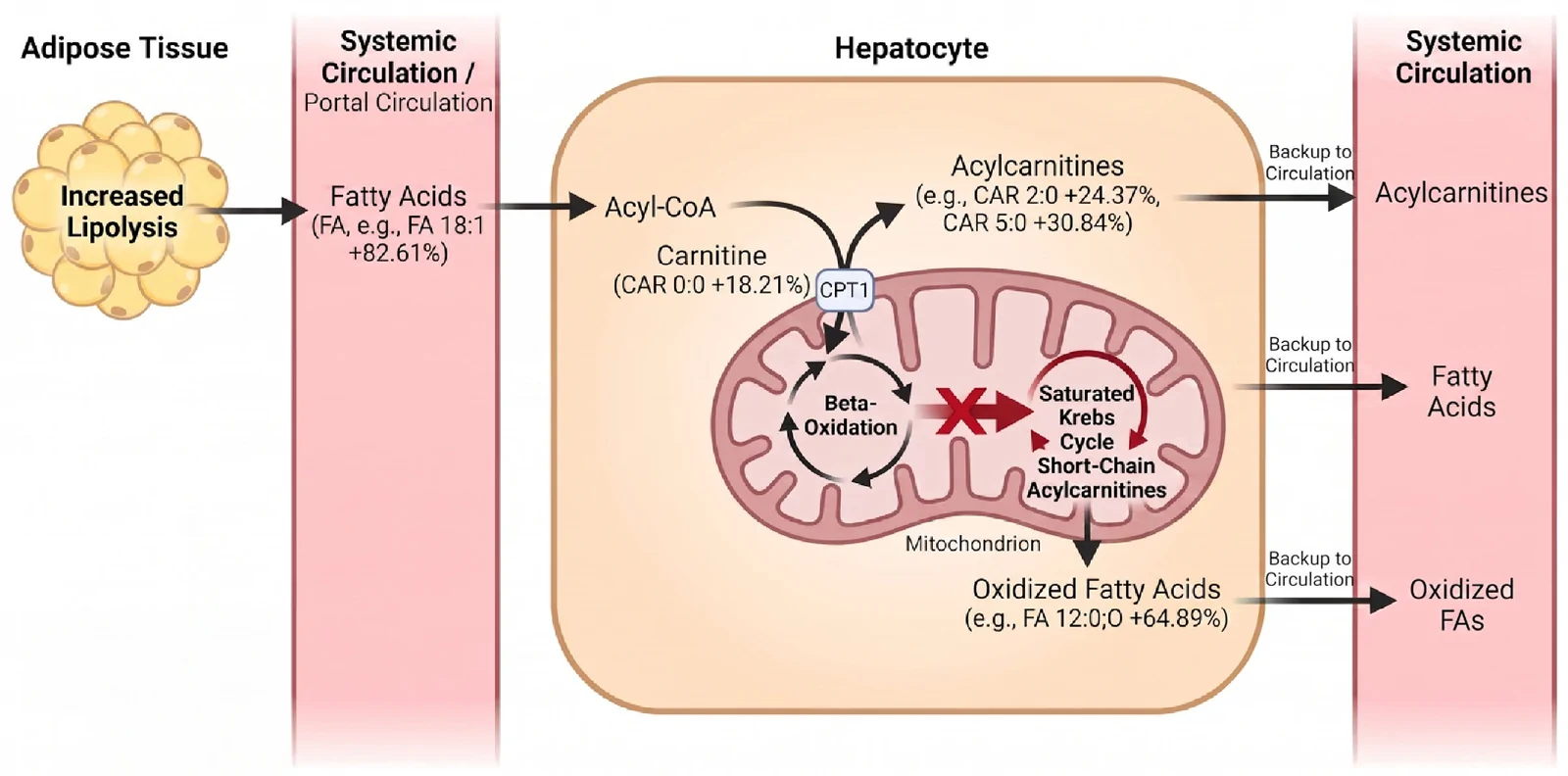
Conceptual model of potential alterations in fatty acid and acylcarnitine metabolism associated with the metabolic profile observed in pediatric MASLD. The proposed changes in mitochondrial fatty acid oxidation and related metabolic pathways were not directly assessed in the present study and therefore represent hypotheses requiring further experimental validation.

Short-chain acylcarnitines are metabolic intermediates whose circulating concentrations can be influenced by fatty acid and amino acid catabolism and may provide indirect information on mitochondrial substrate metabolism. The acylcarnitine pattern observed in the present study may therefore be consistent with alterations in mitochondrial substrate utilization and fatty acid oxidation (Figure 6). Previous studies have suggested that increased circulating acylcarnitines may accompany incomplete fatty acid oxidation or altered mitochondrial substrate utilization [35].

Multiple free fatty acids and their conjugated or oxidized forms were significantly increased, indicating substantial alterations in fatty acid metabolism:

- FA 18:1 increased by 82.61%;
- Oxidized FA (e.g., FA 12:0;O, FA 14:0;O) were also increased, with FA 12:0;O increasing by 64.89%, and FA 14:0;O increasing by 61.36%;
- Other FA species, including FA 18:2 and oxidized forms of FA 18:0, also showed increases ranging from +25.60% to +47.01%;
- 7-oxo-8-amino-nonanoic acid (a fatty acid conjugate) increased by 26.16%.

Taken together, the increases in free fatty acids, oxidized fatty acid species, free carnitine, and short-chain acylcarnitines indicate alterations in systemic lipid and fatty acid metabolism in pediatric MASLD. These changes may be compatible with altered fatty acid mobilization, mitochondrial substrate utilization, and fatty acid oxidation. Nevertheless, metabolomic profiling alone cannot establish increased lipolysis, incomplete fatty acid oxidation, mitochondrial overload, or mitochondrial dysfunction. Further studies incorporating direct functional assessment of mitochondrial metabolism are required to determine the biological mechanisms underlying the observed metabolic signature.

### 3.3. Uric Acid and Metabolic Stress

Uric acid (UA) was elevated by 21.24% in children with MASLD compared with controls. Elevated circulating UA levels have previously been associated with MASLD, including in non-obese younger populations [36]. Previous studies have also linked increased UA with dietary factors, particularly high fructose intake. Fructose metabolism may increase hepatic purine degradation and consequently contribute to increased UA production. Experimental and clinical studies have further implicated UA in metabolic pathways related to oxidative stress, inflammation, and de novo lipogenesis. However, dietary intake, mitochondrial function, inflammatory signaling, and de novo lipogenesis were not directly assessed in the present study. Therefore, the observed increase in UA should be interpreted as part of the metabolic profile associated with pediatric MASLD rather than as evidence of a specific causal mechanism [36,37].

### 3.4. Glycerophospholipid Profiles and Phospholipid Homeostasis

Inositol derivatives (PI/LPI) were upregulated, while choline (LPC/PC) and ethanolamine (LPE) species were predominantly downregulated. This pattern indicated broad alterations in glycerophospholipid metabolism in pediatric MASLD and may reflect changes in membrane lipid remodelling and signaling.

- All lysophosphatidylcholine (LPC) species listed in Table 1 decreased in children with MASLD compared with controls. The changes ranged from 19.58% for LPC 17:1 to 37.87% for LPC 16:0;O, with notable decreases also observed for LPC 18:2 (33.61%) and LPC 20:5 (30.18%);
- Similar to LPCs, all lysophosphatidylethanolamine (LPE) species listed in Table 1 were downregulated in the MASLD group. LPE 16:0 showed the largest decrease (33.90%), while decreases in LPE 18:1, LPE 18:2, LPE 20:4, and LPE 22:6 ranged from 22.59% to 26.80%;
- In contrast to LPC and LPE species, the detected glycerophosphoinositol species (PI and LPI) increased in the MASLD group. PI 39:10;O showed the largest relative change among the metabolites identified in the dataset (+142.56%), while LPI 16:1 and LPI 20:3 increased by 77.74% and 31.02%, respectively.

The dysregulation of phospholipids is a prominent feature of the metabolic profile of pediatric MASLD and may reflect alterations in membrane lipid remodelling and phospholipid homeostasis, as well as altered hepatic lipid handling. Consistent with literature, the cohort showed reductions in multiple phosphatidylcholines (PCs), such as PC 19:2;O (−12.25%) and PC 22:5-3OH (−22.18%). PCs are vital for maintaining membrane fluidity and are important for triglyceride export via very-low-density-lipoprotein. These reductions may therefore be consistent with altered hepatic lipid handling.

However, the findings regarding lysophosphatidylcholines (LPCs) present a notable divergence from key mechanistic literature. LPCs are typically generated during inflammation when phospholipase A_2_ (PLA_2_) degrades PCs. Thus, increased levels of specific LPC species have been associated with inflammatory and lipotoxic processes in metabolic dysfunction-associated steatohepatitis.

The universal decrease in LPCs observed in our cohort may therefore reflect a distinct metabolic pattern in pediatric MASLD. Rather than widespread, active PLA_2_-mediated membrane degradation, this depletion may suggest enhanced LPC clearance (e.g., accelerated conversion to other lipid mediators, such as lysophosphatidic acid) or increased re-acylation back into PCs via the Lands cycle. However, these mechanisms were not directly assessed in the present study. Regardless of the specific enzymatic processes involved, the concurrent depletion of PCs, LPCs, and LPEs suggests broad alterations in glycerophospholipid homeostasis.

### 3.5. Alterations in Amino Acid and Nitrogenous Compounds

- Creatine was upregulated by 72.30%, while creatinine was markedly downregulated by 30.87%;
- Tryptophan was decreased by 14.86%, whereas indoline, an organoheterocyclic compound, was increased by 30.05%;
- Organic acids: malic acid, an intermediate of the tricarboxylic acid (TCA) cycle, was decreased by 17.49%;
- Other nitrogenous compounds: myristoylglycine, a carboxylic acid derivative, was substantially increased by 43.03%.

The analysis revealed an intriguing divergence in the creatine metabolism. A recent integrative metabolomic study in pediatric MASLD reported elevated fecal creatinine levels associated with hepatic dysfunction [38]. In our cohort, however, serum creatinine was decreased by 30.87%. The apparent discrepancy may partly reflect differences in the biological matrix and study population. Since circulating creatinine levels are strongly influenced by age, muscle mass, and renal clearance in growing children, the observed decrease may also be affected by differences in body composition (e.g., altered muscle-to-fat ratio) between the MASLD and healthy control groups, rather than reflecting a MASLD-specific metabolic effect.

Tryptophan metabolism may also be relevant to gut-liver axis-related processes. In our cohort, tryptophan was decreased by 14.86%. Previous studies have shown that tryptophan metabolites derived from host and microbial metabolism can influence intestinal and inflammatory processes. Protective indole derivatives produced by gut microbial metabolism have been associated with maintenance of intestinal barrier integrity [39]. Interestingly, indoline, a reduced indole derivative, was increased in the present study. Although the biological significance of indoline remains unclear, the opposite changes in tryptophan and indoline may indicate alterations in tryptophan-related metabolism.

Finally, alterations in nitrogenous and organic acids may be consistent with changes in mitochondrial and intermediary metabolism. The substantial increase in myristoylglycine (+43.03%), an acylglycine, parallels the observed increase in acylcarnitines, while malic acid, an intermediate of the tricarboxylic acid (TCA) cycle was decreased by 17.49%. These findings may suggest alterations in fatty acid and mitochondrial substrate metabolism; however, mitochondrial function and TCA cycle activity were not directly assessed. Therefore, these observations require further experimental validation.

### 3.6. Comparison of Metabolic Alterations Across Pediatric and Adult MASLD Cohorts

While direct comparison with the existing pediatric MASLD literature is complicated by differences in study populations and analytical protocols, several findings are consistent across studies. Khusial et al. [40] identified several metabolites associated with pediatric NAFLD, including LysoPC 18:1 and LysoPE 20:0, supporting the involvement of lysophospholipid metabolism. This is consistent with the broad reduction in LPC and LPE species observed in our cohort. In addition, Chae et al. [41] reported decreased LPC 18:2 and increased valerylcarnitine (CAR 5:0) in children with NAFLD, paralleling both the lysophospholipid depletion and the increase in short-chain acylcarnitines observed in the present study. Mann et al. [42] also demonstrated pronounced disturbances in phosphatidylcholine-related lipid metabolism in pediatric NAFLD. However, while our study revealed a broad decrease in circulating LPCs, Mann et al. reported increases in selected saturated LPC species, indicating that although glycerophospholipid metabolism is consistently affected, the direction of changes in individual lipid species may vary between cohorts. Differences in cohort characteristics may partly explain these discrepancies. In particular, the non-NAFLD group in Khusial et al. [40] included a high proportion of participants with overweight or obesity, whereas our control group consisted predominantly of healthy, non-obese children. Since BMI was significantly higher in our MASLD group than in controls, some of the observed metabolic alterations may therefore reflect the combined effects of adiposity and MASLD rather than hepatic steatosis alone.

Comparison with adult cohorts similarly suggests substantial agreement at the pathway level but differences in specific metabolite patterns. Adult metabolomic studies have repeatedly identified disturbances in energy metabolism, fatty acid metabolism, phospholipids, oxidative stress, and bile acid homeostasis [43,44,45]. Consistent with these observations, our pediatric cohort showed alterations in TCA-cycle-related metabolites, glycerophospholipids, oxidized fatty acids, and bile acids. Bile acid metabolism provides a particularly illustrative example. Several adult studies have reported increased circulating primary and conjugated bile acids, particularly in more advanced NAFLD/NASH; Masarone et al. [44], for example, observed increasing glycocholic and taurocholic acid concentrations from simple steatosis through NASH to NASH-related cirrhosis. In contrast, our pediatric cohort showed decreased levels of several conjugated primary bile acids. However, pediatric studies also demonstrate considerable heterogeneity. Jahnel et al. [28] reported lower total serum bile acid concentrations in children with nonfibrotic NAFLD, largely due to reduced glycine-conjugated bile acids, whereas children with fibrosis had higher concentrations than those without fibrosis. Lu et al. [46] described increased unconjugated primary bile acids but decreased levels of several secondary bile acids. Thus, bile acid dysregulation appears to be a common feature of MASLD, while the direction of individual bile acid changes may depend on disease stage and cohort characteristics.

These differences likely reflect both biological and methodological heterogeneity. Age and pubertal status, duration and severity of liver disease, obesity, insulin resistance, dietary factors, and differences in control-group composition may all influence circulating metabolite profiles. In addition, the analytical platform, chromatographic separation, sample preparation, and metabolite coverage may contribute to between-study variability. This interpretation is supported by Mann et al. [42], who showed that most lipid species associated with histological NAFLD severity in children did not show comparable associations in adults, despite broader similarities in the affected lipid pathways. Our marked increase in 12α-hydroxy-3-oxocholadienic acid may represent an additional bile-acid-related alteration; however, given the limited information on this metabolite in MASLD, its biological significance requires independent validation. Overall, our findings support the presence of shared metabolic disturbances in pediatric and adult MASLD, particularly involving glycerophospholipid metabolism, fatty acid handling, energy metabolism, oxidative lipid metabolism, and bile acid homeostasis, while suggesting that individual metabolite signatures may vary according to age, adiposity, and disease stage.

### 3.7. Limitations

This study has several limitations that should be considered when interpreting the results. First, the relatively small sample size and single-center design may limit the generalizability of the findings to broader pediatric MASLD populations. Second, although untargeted metabolomics enabled comprehensive metabolic profiling, the annotated metabolites were not validated in an independent external cohort. Third, the cross-sectional nature of the study precludes conclusions regarding causality between the observed metabolic alterations and disease progression. In particular, the associations between altered bile acid metabolism, intestinal barrier dysfunction, and mitochondrial impairment should be interpreted as biologically plausible hypotheses rather than direct mechanistic evidence.

Furthermore, gut permeability, microbiome composition, splenic immune activity, inflammatory mediators, hepatic BA synthesis and conjugation, BA transporter activity, and FXR–FGF19 signaling were not directly assessed. Consequently, the present data cannot distinguish upstream hepatic or liver–spleen-mediated disturbances from primary microbial alterations or determine their relative contributions to the observed serum BA profile.

Finally, despite the robust discrimination achieved by multivariate analyses, the possibility of model overfitting cannot be completely excluded and should be addressed in future studies involving larger cohorts and independent validation datasets. Nevertheless, the consistency of the observed metabolomic alterations with previously reported mechanisms of pediatric MASLD supports the biological relevance of the identified signatures. Additionally, the MASLD and control groups differed significantly in BMI, which represents an important potential confounding factor. Therefore, some of the observed metabolomic alterations may be related to obesity or increased adiposity rather than MASLD-specific hepatic changes, and their independent contributions cannot be distinguished in the present study. Future studies involving BMI-matched control groups or statistical adjustment for adiposity-related variables are warranted to better distinguish MASLD-associated metabolic alterations from those related to obesity.

## 4. Materials and Methods

### 4.1. Study Population

Twenty-one patients with MASLD were enrolled in the study. The diagnosis of MASLD was established based on overweight/obesity, elevated transaminases, and liver steatosis confirmed by ultrasound and transient elastography. Other causes of liver steatosis, such as infectious and metabolic diseases, were excluded. The patients were recruited from the outpatient liver clinic at The Children’s Memorial Health Institute in Warsaw, Poland. The control group consisted of twenty-two participants in whom risk factors for liver disease were excluded based on clinical and laboratory data, and were matched to the MASLD group by age and sex. However, as expected by the study design, the control group differed significantly from the MASLD group regarding BMI (*p*-value < 0.05, Mann-Whitney U-test). The detailed demographic, clinical, and laboratory characteristics of the study groups are summarized in Table 2.

### 4.2. Sample Collection

Blood samples were collected in the morning under fasting conditions from all patients and healthy controls. All participants reported no use of medications or dietary supplements at the time of sample collection. All blood samples were collected using collection tubes from the same manufacturing lot and according to the same standardized procedure.

### 4.3. Stock Solutions and Calibration Standards

In the sample preparation procedure, methanol, water and acetonitrile were used (POCh S. A., Gliwice, Poland). Internal Standard (IS)—Rotenone was purchased from Sigma-Aldrich (St. Louis, MO, USA). Acetonitrile, formic acid (FA) (Honeywell, Sigma-Aldrich, Gillingham, Dorset, UK) and water (POCh S.A., Gliwice, Poland) were used to prepare the mobile phases. The stationary phase used was ACE C18 column (150 × 2.1 mm, 2.6 μm; Advanced Chromatography Technologies, Aberdeen, Scotland, UK).

### 4.4. Sample Preparation

The preparation of serum samples for metabolomic analysis was performed based on the previously described protocol, with minor modifications [47]. All serum samples were initially stored at –80 °C and, when applicable, were transferred to –20 °C approximately 24 h prior to analysis to allow gradual temperature equilibration. On the day of analysis, samples were thawed on ice for approximately 2 h.

Protein precipitation and metabolite extraction were carried out using a methanol-based solution. A mixture of methanol cooled to –20 °C and containing 1 ppm of the internal standard (IS), rotenone, was prepared in a 3:1 ratio relative to the serum volume. Aliquots of 300 µL of this MeOH/IS mixture were dispensed into pre-labelled Eppendorf tubes kept in an ice-cooled container. Subsequently, 100 µL of serum was added to each tube containing the methanol mixture, resulting in a total volume of 400 µL per sample.

The mixtures were vortexed for 1 min to ensure thorough mixing and then placed back on ice for 10 min to facilitate protein precipitation. Samples were centrifuged at 21,000× *g* for 10 min at 4 °C. Following centrifugation, 250–300 µL of the clear supernatant was carefully transferred into chromatographic vials equipped with inserts.

To prepare the quality control (QC) sample, 20 µL from each individual sample was pooled into a single Eppendorf tube prior to extraction. The pooled QC sample was processed using the same sample preparation procedure as the study samples and transferred into a separate vial. The pooled QC sample was injected five consecutive times at the beginning of the analytical sequence to condition the analytical system and subsequently every six study samples to monitor analytical stability. All study samples were analyzed within a single analytical batch and the injection order was randomized to minimize potential order-related bias. No additional QC-based signal drift or batch correction was applied, as the regularly injected QC samples demonstrated consistent analytical performance throughout the sequence. A blank sample consisting of the MeOH/IS mixture alone was prepared and processed in parallel to serve as a negative control. 5 μL of an aliquot was subjected to LC–MS/MS analysis.

### 4.5. Apparatus and Chromatographic Conditions

An Agilent 1260 Infinity LC system coupled with an Agilent 6540 UHD Quadrupole Time-of-Flight Accurate-Mass Mass Spectrometer (Agilent, Santa Clara, CA, USA) was used. Electrospray ionization in a positive and negative mode was applied. Ion source parameters were as follows: gas temperature 300 °C, gas flow 8 L/ min., nebulizer 45 psi, sheath gas temperature 350 °C, sheath gas flow 11 L/min., capillary 3500 V, nozzle voltage 1000 V, fragmentor 130 V. The mobile phase was water with 0.1% FA as a phase A and acetonitrile with 0.1% FA as a phase B. The chromatographic conditions were as follows: run time 20 min., flow rate 0.50 mL/min., gradient elution mode, injection volume 5 μL, column oven temperature 40 °C. The gradient profile was as follows: 0 min. 5% B; 2 min. 5% B; 10 min. 70% B; 13 min. 98% B; 17 min. 98% B; 17.20 min. 5% B, 20 min. 5% B. The controlling software for the LC–MS/MS system was LC/MS Data Acquisition Agilent MassHunter Workstation Software (version B.08.02). Data processing and quantitation were made using Quantitative Analysis MassHunter Workstation Software (version B.08.00).

### 4.6. Data Analysis

#### 4.6.1. LC-MS Data Treatment

The quality of the analysis was assessed using Agilent MassHunter Quantitative Analysis software (version B.08.00) through the examination of total ion chromatograms (TIC), evaluation of the pressure curves to verify the stability of chromatographic conditions, and assessment of the stability of the reference mass signals across all samples, QCs and blanks. All samples satisfied the established quality assessment criteria.

The collected raw data were processed to remove background noise and unrelated ions using Molecular Feature Extraction (MFE) with recursion in MassHunter Profinder software (version 10.0.2, Build 10.0.2.162, Agilent Technologies). MFE generated a list of putative molecular features defined by their mass, retention time (RT), and abundance. For both positive and negative ion modes, the threshold for background noise in data extraction was set to 600 counts. To identify co-eluting adducts of the same feature, the following adduct settings were applied: +H, +Na, +K, +CHNaO_2_, and +C_2_H_2_Na_2_O_4_ in positive ion mode, and −H, +HCOO, +Cl, +C_2_HF_3_O_2_, and +C_3_H_2_F_3_NaO_4_ in negative ion mode. Neutral losses of C_2_H_4_O_2_ and CH_3_ were considered in negative ion mode, while water loss was included in both polarities. The maximum permitted charge state was set to two.

Alignment of features was performed based on *m*/*z* and RT similarities across the samples. The applied parameters were 1% and 0.20 min for the RT window, and 20 ppm and 2 mDa for mass tolerance. These parameters were selected following an assessment of the raw data. The resulting RT and mass data pairs were used as input criteria for recursive analysis. For this targeted search, the feature finding and alignment parameters were identical to those used during the initial MFE step. All extracted peaks were manually curated and corrected where necessary. A final list of ions was generated, characterized by their exact monoisotopic mass, RT, and peak area.

Prior to statistical analysis, data were filtered to retain only metabolic features detected in >80% of QC samples and with a Relative Standard Deviation (RSD) of signals in QC samples below 20%. Additionally, only features present in at least 50% of the samples in at least one of the biological groups under comparison were retained. To reduce technical variation related to differences in total signal intensity across samples, data were normalized to the total useful signal (TS), defined as the sum of the intensities of high-confidence metabolic features. The normalization strategy was selected based on a comparison of non-normalized data, total useful signal-normalized data, and internal standard-normalized data. The performance of the different approaches was evaluated using PCA, with particular attention to the clustering and dispersion of QC samples; TS normalization provided the most consistent QC clustering and was therefore selected for subsequent analyses. Missing values were imputed according to the procedure described by Armitage et al. [48].

#### 4.6.2. Statistical Analysis

To assess data quality, multivariate statistical analysis was performed using SIMCA-P + 13.0.3.0 (Umetrics, Umeå, Sweden). Principal Component Analysis (PCA) was employed to visualize the clustering of QC samples (Figure 1A,B), whereas Orthogonal Partial Least Squares Discriminant Analysis (OPLS-DA) was used to visualize the discrimination between study groups (Figure 1C,D). The quality of the OPLS-DA models was evaluated using R^2^Y, representing the proportion of variation in the response variable (Y) explained by the model, and Q^2^, reflecting its predictive ability estimated by cross-validation. The statistical significance of the OPLS-DA models was assessed using cross-validated analysis of variance (CV-ANOVA), with *p*-value < 0.05 considered statistically significant.

To evaluate the differences between the study groups, univariate statistical analysis was performed. The non-parametric Mann–Whitney U-test with Benjamini-Hochberg false discovery rate (FDR) correction was applied, with statistical significance set at 95% (*p*-value < 0.05). This analysis was conducted in MATLAB (version 2020a; MathWorks, Natick, MA, USA) using in-house scripts. Differences between the groups were expressed as percentage changes, calculated based on the median values of each metabolite within the respective groups.

#### 4.6.3. Metabolite Annotation

Features selected during univariate statistical analysis were identified based on tandem mass spectrometry (MS/MS) fragmentation. MS/MS analyses were conducted under chromatographic conditions identical to those used in the primary analysis. Using the previously determined accurate masses and retention times, ions were targeted for collision-induced dissociation (CID) fragmentation on the fly. The METLIN, KEGG, LIPIDMAPS, and HMDB databases were accessed via the CEU Mass Mediator (http://ceumass.eps.uspceu.es/mediator; accessed on 30 July 2025) and used to search accurate masses of metabolite candidates, using a mass tolerance of ±20 ppm. All reported metabolites were further evaluated using MS/MS data. Metabolite identities were subsequently annotated by matching the experimental MS/MS spectra against reference spectra available in the HMDB database, using a Fit score cut-off of ≥0.7. Due to the very limited number of MS/MS spectra available in the databases, lipids underwent manual annotation, with lipid structural elucidation based on established lipid fragmentation patterns [49,50,51,52,53].

## 5. Conclusions

This study shows that pediatric MASLD is associated with a distinct serum metabolomic profile characterized by alterations in several metabolic domains, particularly lipid and fatty acid metabolism, bile acid and sterol metabolism, and amino acid-related pathways. The observed changes indicate broad metabolic perturbations in pediatric MASLD and identify metabolic signatures that may contribute to a better characterization of the disease phenotype and warrant further investigation as candidate biomarkers.

These findings provide a basis for future validation and mechanistic studies. Further research in larger, independent cohorts, together with targeted validation and complementary functional approaches, will be important to determine the specificity, clinical relevance, and biological significance of the identified metabolic signatures.

## Figures and Tables

**Table 1:** Serum metabolites showing significant differences between children with MASLD and controls. Abbreviations: CAR: carnitine; FA: fatty acids; LPC: lysophosphatidylcholine; PC: phosphatidylcholine; LPE: lysophosphatidylethanolamine; LPI: lysophosphatidylinositol; PI: phosphatidylinositol; ST: sterol lipids. A positive value means that an abundance of a particular metabolite was higher, whereas a negative value indicates that abundance was lower in the MASLD group vs. the control group. Significance was evaluated using the Mann-Whitney U-test with a 95% confidence level. The table presents the complete list of annotated metabolites showing statistically significant differences between studied groups (FDR-adjusted *p*-value < 0.05, Benjamini-Hochberg correction).

Metabolite	% of Change	*p*-Value	*p*BH
Creatine	+72.30	0.0083	0.0380
Creatinine	−30.87	0.0029	0.0199
Indoline	+30.05	0.0095	0.0400
Malic acid	−17.49	0.0068	0.0420
Myristoylglycine	+43.03	0.0004	0.0098
Tryptophan	−14.86	0.0054	0.0398
12α-hydroxy-3-oxocholadienic acid	+118.58	0.0004	0.0095
Uric acid	+21.24	0.0059	0.0312
CAR 0:0	+18.21	0.0025	0.0186
CAR 2:0	+24.37	0.0054	0.0296
CAR 3:0	+34.25	0.0014	0.0129
CAR 5:0	+30.84	0.0035	0.0230
7-oxo-8-amino-nonanoic acid	+26.16	0.0115	0.0464
FA 12:0;O	+64.89	0.0071	0.0420
FA 14:0;O	+61.36	0.0001	0.0159
FA 16:0;O	+39.76	0.0019	0.0238
FA 18:0;O	+31.16	0.0029	0.0297
FA 18:0;O2	+25.60	0.0068	0.0420
FA 18:1	+82.61	0.0031	0.0314
FA 18:1;O	+35.34	0.0071	0.0420
FA 18:2	+47.01	0.0032	0.0324
LPC 16:0	−22.46	<0.0001	0.0003
LPC 16:0;O	−37.87	<0.0001	0.0016
LPC 17:1	−19.58	0.0048	0.0272
LPC 18:1	−22.73	<0.0001	0.0001
LPC 18:2	−33.61	<0.0001	0.0001
LPC 18:3	−29.81	<0.0001	0.0007
LPC 20:4	−27.59	0.0008	0.0086
LPC 20:5	−30.18	<0.0001	0.0003
PC 19:2;O	−12.25	0.0026	0.0292
PC 22:5-3OH	−22.18	0.0006	0.0114
LPE 16:0	−33.90	0.0005	0.0057
LPE 18:1	−22.59	0.0074	0.0350
LPE 18:2	−26.80	0.0016	0.0141
LPE 20:4	−22.74	0.0018	0.0154
LPE 22:6	−24.43	0.0054	0.0296
LPI 16:1	+77.74	0.0092	0.0482
LPI 20:3	+31.02	0.0092	0.0482
PI 39:10;O	+142.56	<0.0001	0.0013
ST 19:1;O2;S	+34.30	0.0063	0.0415
ST 22:1;O3;S	+84.77	0.0003	0.0095
ST 21:3;O5	−40.61	0.0002	0.0024
ST 24:1;O3;Gly	−54.55	0.0048	0.0384
ST 24:1;O3;Tau	−62.47	0.0083	0.0453
ST 24:1;O4;Gly	−54.82	<0.0001	0.0018
glycochenodeoxycholic acid 7-sulfate	−60.76	0.0024	0.0286

**Table 2:** Demographic, clinical and laboratory characteristics of the study groups. Abbreviations: BMI: body mass index; NA: not applicable; ALT: Alanine aminotransferase; AST: Aspartate aminotransferase; GGTP: Gamma-glutamyl transferase; CAP: Controlled Attenuation Parameter; E: Liver Stiffness Measurement. Sex distribution is presented as percentages, while the other characteristics are expressed as median (min–max).

Clinical and Anthropometric Parameters	Patients with MASLD	Control Group
Demographics		
Sex (Male/Female)	71/29	64/36
Age [years]	14 (9–17)	15 (12–18)
Anthropometry		
BMI [kg/m^2^]	28 (23–39)	21 (15–30)
BMI z-score	2.15 (0.86–3.10)	NA
Waist circumference [cm]	91 (76–109)	NA
Biochemistry		
ALT [IU/L]	27 (12–123)	7 (3–17)
AST [IU/L]	26 (14–60)	15 (12–30)
GGTP [IU/L]	21 (11–121)	NA
Transient Elastography		
CAP [dB/m]	294 (228–390)	NA
E [kPa]	6 (3–11)	NA

## Data Availability

The original contributions presented in this study are included in the article or in the Supplementary Materials. Further inquiries can be directed to the corresponding author.

## Supplementary Materials

The following supporting information can be downloaded at: https://www.mdpi.com/article/10.3390/ijms27198742/s1.

## Abbreviations

The following abbreviations are used in this manuscript:

ASBT	apical sodium-dependent bile acid transporter
BA	bile acid
BMI	body mass index
CAR	carnitine
CID	collision-induced dissociation
CYP7A1	cholesterol-7-α-hydroxylase
DNL	de novo lipogenesis
FA	fatty acids
FA	formic acid
FDR	false discovery rate
FGF19	fibroblast growth factor 19
FXR	farnesoid X receptor
IBABP	ileal bile acid binding protein
IS	internal standard
LC-MS	liquid chromatography-mass spectrometry
LPC	lysophosphatidylcholine
LPE	lysophosphatidylethanolamine
LPI	lysophosphatidylinositol
LPS	lipopolysaccharide
MASH	metabolic dysfunction-associated steatohepatitis
MASLD	metabolic dysfunction-associated steatotic liver disease
MFE	molecular feature extraction
OPLS-DA	orthogonal partial least squares discriminant analysis
PC	phosphatidylcholine
PCA	principal component analysis
PI	phosphatidylinositol
PLA2	phospholipase A_2_
QC	quality control
QTOF	quadrupole time of flight
ROS	reactive oxygen species
RSD	relative standard deviation
RT	retention time
RXR	retinoid X receptor
ST	sterol lipids
TCA	tricarboxylic acid
TS	total useful signal
UA	uric acid
VLDL	very-low-density lipoprotein
